# Editorial and Miscellaneous

**Published:** 1866-03

**Authors:** 


### EDITORIAL AND MISCELLANEOUS.

Again, after over four years absence of the Journal, we, under embarrassing circumstances however, reproduce it, and hope to be able, by the realization of moderate expectations of patronage, to furnish the medical reader with a periodical worthy of his encouragement and useful to him as a practitioner.

In the conduct of the Journal, free discussions on all scientific subjects will be allowed and encouraged, but no vituperation or personal abuse will be admitted to its pages. That course of moderation and independence which formerly governed its editorial management, will again characterize the Journal.

The system of medical ethics, embraced in "the golden rule," "Do unto others as you would have them do to you," will be observed and odvocated.

As the exponent of the system of medical education adopted by the Atlanta Medical College, thorough instruction in all the medical sciences, and a proper understanding of them by the student, in order to admission to the degree, will be earnestly advocated.

While no captious objections will be made to the course pursued by sister Institutions, yet in the fearless advocacy of principles upon which the honor and usefulness of the profession and the public welfare depend, we will stand or fall. No party cliques or personal preferences shall swerve us from the path of rectitude, honor and usefulness. Institutions and Journals engaged in the great and philanthropic cause will not be viewed as rivals and marked as objects of distrust, malevolence, or envy, but will be recognized as colaborers in the advancement of medical science.

Circumstances beyond our control have delayed the publication of the first number several weeks beyond the time designated in the published prospectus; and the pecuniary embarrassments which, since the close of the war, interferes materially with all enterprises requiring money, have ob-obliged us to curtail the number ot pages originally intended. It will be seen, however, that we have correspondingly reduced the subscription price. The paucity of this number in original communications will doubtless be excused by our readers when they remember that the uncertainty of a forthcoming work of this kind discourages contributors in the preparation of articles till the Journal becomes "a fixed fact" by the issue of the "first number." To this department we attach the highest importance, and shall make early arrangements to secure contributors whose experience during the late war, and whose scientific attainments will insure articles unusually attractive and useful. The great dearth in medical literature during the past four years from the complete arrest, through the South, of the channels of communication, leaves in store upon the records and diaries of army surgeons and private practitioners, large accumulations of valuable information to the medical profession.

We desire to make our Journal a medium of communication to those who have at heart the advancement of our philanthropic profession. And we solicit contributions from those who have a disposition to write, either as *sporadic* communications or in the form of a series of articles reported monthly.

The small number of Medical Journals revived in the South since the war, and the inability as yet to obtain in exchange foreign, or but a limited number of Northern journals, confine our "Selections" to but few journals. We hope, however, soon to be able to give important items from all journalism.

As an advertising medium we expect, by giving it a pretty general circulation in Georgia, Alabama and Florida, with a more limited number in the States of North and South Carolina, Tennessee, Mississippi, Arkansas, Louisiana and Texas, to make the Atlanta Medical & Surgical Journal equal for this purpose to any publication of the kind. Our rates of advertising, as will be seen by reference to PROSPECTUS in this number, are as moderate as the times will justify, and it is important to us in the fulfillment of our engagements for the publication of the Journal that payments be made promptly. We, more than heretofore, feel the necessity of requiring prompt cash payments for subscriptions, as well as advertisements. The amounts taken separately are inconsiderable, and may be met by the subscriber or advertiser without inconvenience, but in the aggregate become very important to the proprietors, and the publication of the Journal itself, without destructive drafts upon resources, that must necessarily be applied elsewhere, depends upon their payment.

